# Supplementary material for: A Fragmenting Protocol with Explicit Hydration for Calculation of Binding Enthalpies of Target-Ligand Complexes at a Quantum Mechanical Level
Source: Int J Mol Sci. 2019 Sep 6;20(18):4384. doi: 10.3390/ijms20184384 (PMC6770515; doi:10.3390/ijms20184384)
Supplement: Supplementary file 1 [file ijms-20-04384-s001.pdf]

Supplementary Information

A fragmenting protocol with explicit hydration for calculation of binding enthalpies of target-ligand complexes at a quantum mechanical level

István Horváth, Norbert Jeszenői, Mónika Bálint, Gábor Paragi, Csaba Hetényi*

Table of Contents

Supplementary Fig. 1 Fragmenter 2

Supplementary Fig. 2 Data flow in Fragmenter 3

Supplementary Fig. 3 Steps of extraction of an example target fragment 4

Supplementary Fig. 4 The frontal web page of Fragmenter (www.fragmenter.xyz) 5

Supplementary Fig. 5 An example Results tab of Fragmenter web service 6

Supplementary Fig. 6 Contents of project_1k1j.inp file 7

Supplementary Table 1 Input parameters of Fragmenter 8

Supplementary Table 2 The effect of inter-residual topological distance 9

Supplementary Table 3 Net charges and experimental thermochemical data of the systems 10

Supplementary Table 4 Raw data of Table 2 (β≠0) 11

Supplementary Table 5 Linear regressions re-calculated without 2ke1 14

Supplementary Table 6 Linear regression (COSMO/Shell 3) re-calculated using two sub-sets 17

Supplementary Table 7 Linear regressions of three models with β=0 18

Supplementary Fig. 1 Fragmenter

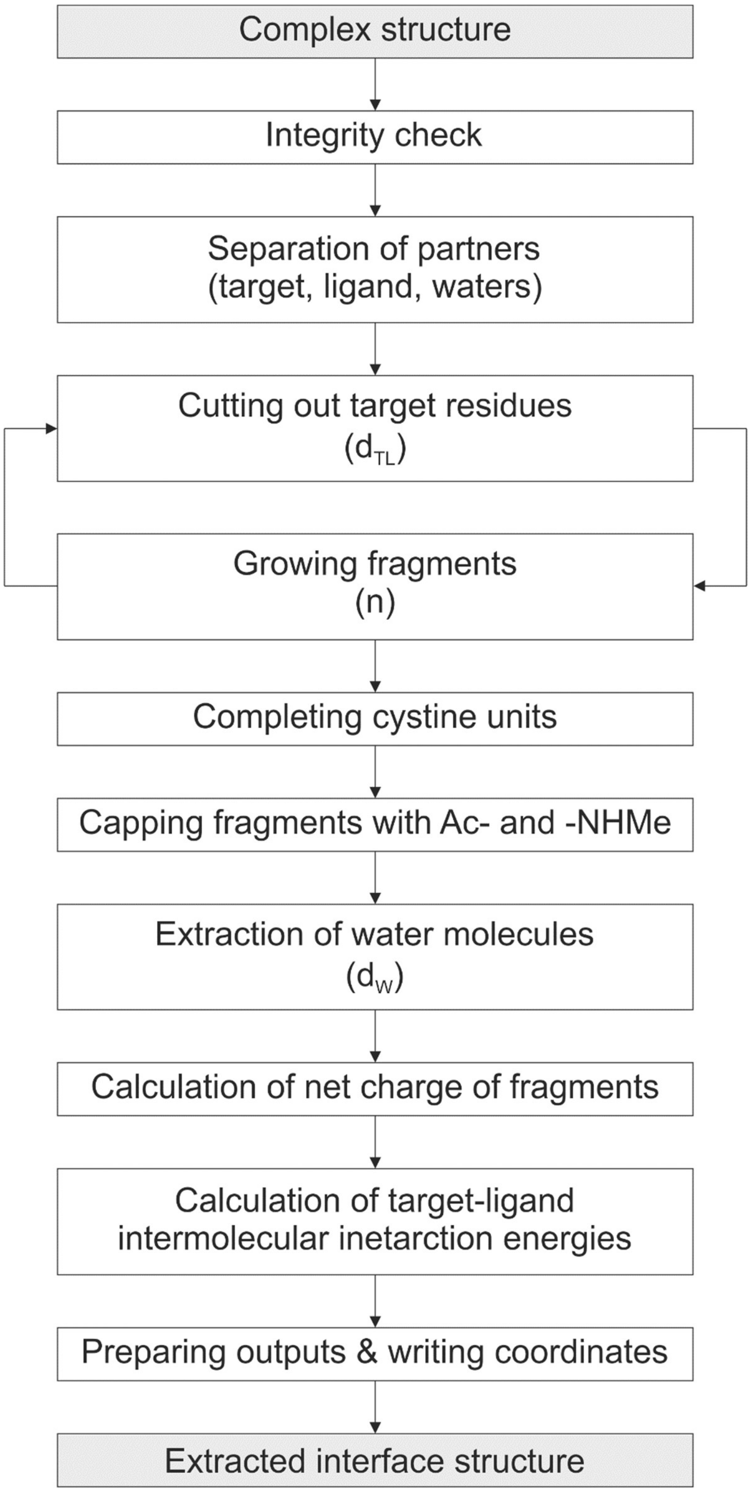

Supplementary Fig. 2 Data flow in Fragmenter

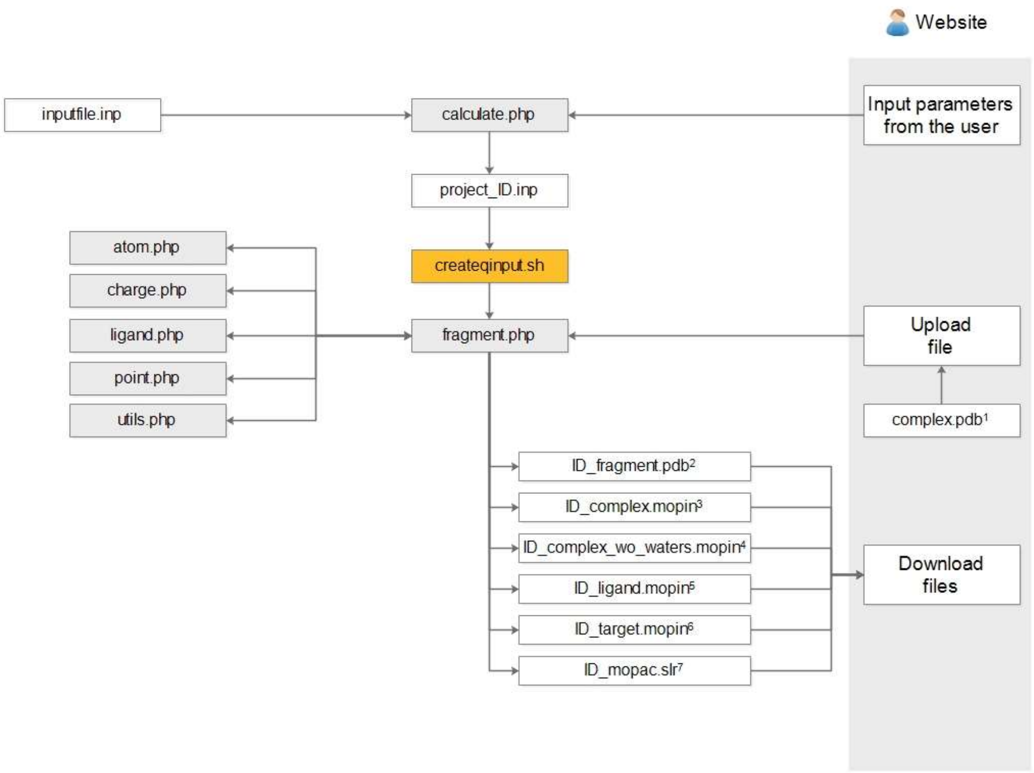

[1]Complex structure uploaded by user
[2]Interface structure PDB
[3]Interface structure Mopac input
[4]Interface structure Mopac input without water molecules
[5]Ligand PDB
[6]Target PDB
[7]SLURM input file

Supplementary Fig. 3 Steps of extraction of an example target fragment from the C-terminal VEFFHVQDL sequence of the target protein of System 2roc.

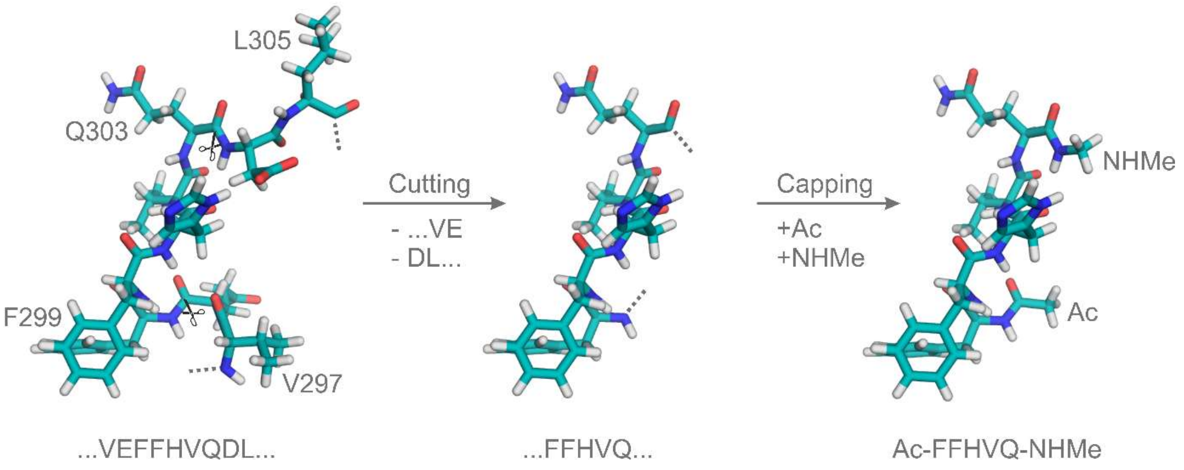

Supplementary Fig. 4 The frontal web page of Fragmenter (www.fragmenter.xyz)

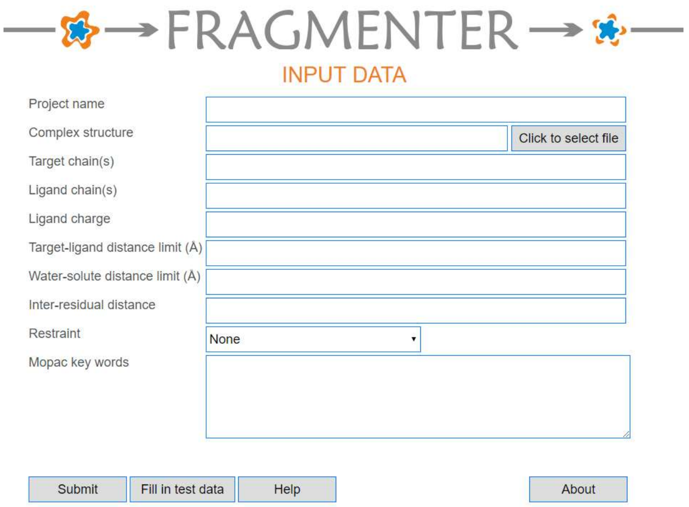

Supplementary Fig. 5 An example Results tab of Fragmenter web service

FRAGMENTER

RESULTS

Input data

PDB ID	1k1i_input.pdb
Project name	Test project
Target chain(s)	A
Ligand chain(s)	B
Ligand charge	0
Target-ligand distance (Å)	5
Water-solute distance (Å)	5
Inter-residual distance	0
Restraint	NONE
Additional mopac parameters	PM7 PREC GRAD EF EPS=78.3 GNORM=1 MMOK GEO-OK DUMP=30M T=4W LET CYCLES=1000000000 MOZYME
All charge	-1
Fragment charge	-1

Interface structure

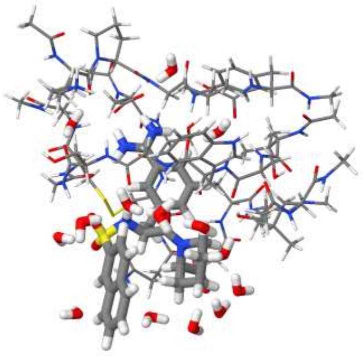

JSmol

Download

Complex structure (PDB)
Interface structure (PDB)
Interface structure (Mopac input)
Interface structure without waters (Mopac input)
Ligand structure (Mopac input)
Target (Mopac input)
Slurm input

Intermolecular interaction energy estimate per target residue

Residue #	Residue name	E_{inter}(LJ)	E_{inter}(Coulomb)	E_{inter}(total)
80	ACE	-0.04	0.59	0.54
81	LEU	-0.87	-0.16	-1.04
82	NME	-0.01	-1.18	-1.19
170	ACE	-0.04	0.93	0.89
171	ASP	1.23	-6.10	-4.87
172	SER	0.07	-12.94	-12.87
173	CYS	-4.42	0.03	-4.39
174	GLN	-7.43	-2.69	-10.12
175	GLY	-0.42	-0.03	-0.45
176	ASP	-0.60	1.12	0.52
177	SER	-1.00	0.22	-0.78
178	NME	-0.05	-1.39	-1.44
190	ACE	-0.03	0.49	0.46
191	VAL	-0.57	1.74	1.17
192	SER	-1.83	-0.29	-2.12
193	TRP	-5.82	-3.34	-9.16
194	GLY	-3.98	2.35	-1.63
195	SER	-2.07	-1.50	-3.56
196	GLY	3.14	-9.39	-6.25
197	CYS	-2.50	4.57	2.07
198	ALA	-0.37	2.31	1.94
199	NME	-0.07	-1.84	-1.90
201	ACE	-0.02	1.16	1.15
202	LYS	-0.43	-2.88	-3.31
203	PRO	-0.55	1.47	0.92
204	GLY	-0.68	5.29	4.61
205	VAL	-0.52	-2.01	-2.53
206	TYR	-0.42	-1.19	-1.61
207	NME	-0.01	-1.50	-1.51
Total		-30.29	-26.16	-56.46

Submit new project | Help

Supplementary Fig. 6 Contents of project_1k1j.inp file

```
# comment: #
# optional: $  e.g. $EPS=78.4
# e.g. @RESTRAINT=CALPHA, RESTRAINT=NONE, RESTRAINT=HEAVY, RESTRAINT=BACKBONE
#METHODS=AM1,PM3, PM6 , PM6-DH+, PM7, RM1              # methods, separated with comma
METHODS=PM7                                             # method
WATERDIST=5                                             # water distance
LIGDIST=5                                               # ligand distance
NEIGHBOUR=0                                             # neighbour number
PDBPATH=/home/user/fragment/uploads/                    # pdb path
PDBNAME=1k1j.pdb                                        # pdb file name
#ALL_IN_VACUUM                                          # create complex, ligand and target in vacuum
#LIGANDCHARGE=-1                                        # ligand charge
#FRAGMENTCHARGE=-1                                      # fragment charge
TARGETCHAINID=A                                         # target chain ID
LIGANDCHAINID=B                                         # ligand chain ID
RESTRAINT=NONE                                          # restraint (N=NONE, H=HEAVY, B=BACKBONE, A=CALPHA)
MOPACPARAMS=PREC GRAD EF GNORM=1 MMOK GEO-OK DUMP=30M T=4W LET CYCLES=1000000000 MOZYME
                                                        # all other mopac parameters
SCRIPTPATH=$HOME/fragment/bin/createmopacinput.sh       # script path
MOPAC_LICENSE=$HOME/bin/mopac2012                       # mopac license path
MOPAC=$HOME/bin/mopac2012/MOPAC2012.exe                 # mopac path
#@RESTRAINT=CALPHA, RESTRAINT=NONE, RESTRAINT=HEAVY, RESTRAINT=BACKBONE      # for list
#@RESTRAINT=CALPHA, RESTRAINT=NONE                      # for list
PROCESSNAME=1k1j.pdb                                    # this name appears in the qsub statistics
PHPPATH=/usr/bin/php                                    # php binary path
```

Supplementary Table 1 Input parameters of Fragmenter

Parameter	Description
Project name	Identifier of the process for the mopac and the output file names
Complex structure	The full path and file name of the input PDB file
Target chain(s)	Target chain identifier(s) as indicated in the PDB file
Ligand chain(s)	Ligand chain identifier(s) as indicated in the PDB file
Ligand charge*	Net charge of the ligand. By default, 0 is set.
Target-ligand distance limit (d_{TL})	Maximal distance between the closest heavy atoms of target and ligand (solute) molecules (Å)
Water-solute distance limit (d_W)	Maximal distance between the closest heavy atoms of water and solutes (target and ligand) (Å)
Inter-residual distance (n)	Parameter n determines the length of the peptide fragments. If $n > 0$, then n connecting amino acid residues are added to the fragment molecule. If $n = 0$ only amino acids with $d_{cls} \leq d_{TL}$ are added to the fragment chain, where d_{cls} is the shortest distance between (the closest) heavy atoms of the partners. If $n = 1$, the sequential first neighbours are also attached to the terminus (termini) of the fragment chain, even if the attached amino acids have a $d_{cls} > d_{TL}$, etc.
Restraint	C-alpha atoms, backbone atoms, heavy atoms can be restrained separately
Mopac key words*	Key words of Mopac including parameterization are copied into the first line of the generated Mopac input file. By default, the following key words are set: PM7 PREC GRAD EF EPS=78.3 GNORM=1 MMOK GEO-OK DUMP=30M T=4W LET CYCLES=1000000000 MOZYME[49]

*Optional.

Supplementary Table 2 The effect of inter-residual topological distance (n) on the selection of target amino acids*

Target sequence	n=0	n=1	n=2	n=3	n=4	n=5
L166						L166
E167					E167	E167
G168				G168	G168	G168
G169			G169	G169	G169	G169
K170		K170	K170	K170	K170	K170
D171	D171	D171	D171	D171	D171	D171
S172	S172	S172	S172	S172	S172	S172
C173	C173	C173	C173	C173	C173	C173
Q174	Q174	Q174	Q174	Q174	Q174	Q174
G175		G175	G175	G175	G175	G175
D176		D176	D176	D176	D176	D176
S177	S177	S177	S177	S177	S177	S177
G178		G178	G178	G178	G178	G178
G179			G179	G179	G179	G179
P180				P180	P180	P180
V181					V181	V181
V182						V182
C183						
S184						
G185						
K186						K186
L187					L187	L187
Q188				Q188	Q188	Q188
G189			G189	G189	G189	G189
I190		I190	I190	I190	I190	I190
V191	V191	V191	V191	V191	V191	V191
S192	S192	S192	S192	S192	S192	S192
W193	W193	W193	W193	W193	W193	W193
G194	G194	G194	G194	G194	G194	G194
S195	S195	S195	S195	S195	S195	S195
G196	G196	G196	G196	G196	G196	G196
C197	C197	C197	C197	C197	C197	C197
A198	A198	A198	A198	A198	A198	A198
Q199		Q199	Q199	Q199	Q199	Q199
K200			K200	K200	K200	K200
N201		N201	N201	N201	N201	N201
K202	K202	K202	K202	K202	K202	K202
P203	P203	P203	P203	P203	P203	P203
G204	G204	G204	G204	G204	G204	G204
V205	V205	V205	V205	V205	V205	V205
Y206		Y206	Y206	Y206	Y206	Y206
T207			T207	T207	T207	T207
K208				K208	K208	K208
V209					V209	V209
C210						C210

*Amino acid residues are listed for System 3PTB_ben, d_{TL}= 5 Å, n = 0…5.

Increasing the value of the inter-residual topological distance (n), increases the length of the continuous fragment chains extracted from the original chain. We found that n=0 was appropriate for our calculations. High n values would obviously result in long QM calculation times.

Supplementary Table 3 Net charges and experimental thermochemical data of the systems

Net charges

System code	Ligand	Target fragments	System (total)
3ptb_ben	1	0	1
3ptb_pme	1	-1	0
3ptb_pam	1	0	1
3ptb_pmo	1	0	1
3ptb_pad	1	-1	0
1k1l	2	0	2
1k1m	1	1	2
1k1i	0	-1	-1
1k1j	1	1	2
1jyr	-2	1	-1
1rlq	2	-3	-1
2ke1	4	-5	-1
2bba	1	2	3
1jgn	2	-1	1
2roc	-2	1	-1

Experimental thermochemical data*

System code	T_1	$\Delta H_b(T_1)$	ΔC_p	T_2	$\Delta H_b(T_2)$	Reference
3ptb_ben	298.25	-4.517	-95.602	298.15	-4.507	[2]
3ptb_pme	298.25	-4.422	-100.382	298.15	-4.412	[2]
3ptb_pam	298.25	-6.429	-124.283	298.15	-6.417	[2]
3ptb_pmo	298.25	-3.752	-100.382	298.15	-3.742	[2]
3ptb_pad	298.25	-2.940	-54.971	298.15	-2.935	[2]
1k1l	298.15	-7.863	-525.813	298.15	-7.863	[4]
1k1m	298.15	-8.222	-611.855	298.15	-8.222	[4]
1k1i	298.15	-10.899	-619.025	298.15	-10.899	[4]
1k1j	298.15	-9.465	-621.415	298.15	-9.465	[4]
1jyr	298.15	-7.94	-146	298.15	-7.94	[6]
1rlq	298.15	-10.2	na	298.15	-10.2	[7]
2ke1	296.15	-9.2	na	296.15	-9.2	[8]
2bba	298.15	-15.5	na	298.15	-15.5	[9]
1jgn	298.15	-14.8	-253.2	298.15	-14.8	[10]
2roc	298.15	-14.3	na	298.15	-14.3	[11]

*T, ΔH_b, and ΔC_p are given in K, kcalmol^{-1}, and calmol^{-1}K^{-1} units, respectively. 1 cal = 4.184 J. T_1 is the temperature of original measurement. All ΔH_b values except that of 2ke1 were converted to/measured at 298.15 K. Conversions of ΔH_b values to T_2 was performed according to Kirchhoff's law $\Delta H_b(T_2) = \Delta H_b(T_1) + \Delta C_p [T_2 - T_1]$. Data in $\Delta H_b(T_2)$ column were used as ΔH_b(exp) during the calculations. Precision of original data at T_1 was kept except values converted from kJmol^{-1}, where three decimal digits are used.

Supplementary Table 4 Raw data of Table 2 (β≠0)

Parameters of Eq. 3

Model	α	Δα*	β	Δβ*
Vacuum/Dry	4.3975×10^{-3}	4.8897×10^{-3}	-7.8349	1.4104
Vacuum/Shell 1	6.0855×10^{-3}	3.6583×10^{-3}	-7.0296	1.3944
Vacuum/Shell 2	4.0942×10^{-3}	2.3105×10^{-3}	-6.7765	1.4465
Vacuum/Shell 3	6.7440×10^{-3}	2.1121×10^{-3}	-5.2456	1.3445
COSMO/Dry	7.9586×10^{-2}	2.1696×10^{-2}	-3.4980	1.6027
COSMO/Shell 1	3.7379×10^{-2}	7.5864×10^{-3}	-4.3391	1.0864
COSMO/Shell 2	1.1187×10^{-2}	4.4373×10^{-3}	-5.9395	1.4008
COSMO/Shell 3	2.1972×10^{-2}	3.7381×10^{-3}	-3.1009	1.1038

*Standard error of regression coefficient.

Vacuum/Dry*

System	$\Delta_f H$(Ligand)	$\Delta_f H$(Target)	$\Delta_f H$(Target:Ligand$[H_2O]_s$)	$\Delta_r H$	ΔH_b(calc)	|ε|
3ptb_ben	178.10	-1490.53	-1396.08	-83.65	-8.20	3.70
3ptb_pme	162.59	-1632.88	-1510.70	-40.41	-8.01	3.60
3ptb_pam	167.30	-1497.83	-1403.59	-73.06	-8.16	1.74
3ptb_pmo	130.65	-1718.48	-1669.42	-81.59	-8.19	4.45
3ptb_pad	136.51	-1672.10	-1705.79	-170.20	-8.58	5.65
1k1l	262.49	-1928.22	-1798.53	-132.81	-8.42	0.56
1k1m	19.38	-1688.03	-1720.73	-52.08	-8.06	0.16
1k1i	-117.59	-1957.77	-2164.68	-89.31	-8.23	2.67
1k1j	-33.07	-1617.02	-1695.03	-44.94	-8.03	1.43
1jyr	-1038.98	-1744.62	-2984.92	-201.32	-8.72	0.78
1rlq	-222.49	-1576.62	-2185.57	-386.47	-9.53	0.67
2ke1	-71.83	-2361.52	-3322.49	-889.14	-11.74	2.54
2bba	-740.01	-2916.44	-3749.75	-93.29	-8.25	7.25
1jgn	-1455.38	-2702.38	-4405.34	-247.57	-8.92	5.88
2roc	-2618.04	-2389.65	-5350.57	-342.88	-9.34	4.96

*(kcal/mol)

Vacuum/Shell 1*

System	$\Delta_f H$(Ligand)	$\Delta_f H$(Target)	$\Delta_f H$(Target:Ligand$[H_2O]_s$)	$\Delta_r H$	ΔH_b(calc)	|ε|
3ptb_ben	178.10	-1490.53	-1478.31	-108.08	-7.69	3.18
3ptb_pme	162.59	-1632.88	-1605.48	-77.39	-7.50	3.09
3ptb_pam	167.30	-1497.83	-1615.82	-111.89	-7.71	1.29
3ptb_pmo	130.65	-1718.48	-1747.77	-102.14	-7.65	3.91
3ptb_pad	136.51	-1672.10	-1818.83	-167.64	-8.05	5.11
1k1l	262.49	-1928.22	-2155.93	-201.20	-8.25	0.39
1k1m	19.38	-1688.03	-2004.46	-104.61	-7.67	0.56
1k1i	-117.59	-1957.77	-2302.42	-111.46	-7.71	3.19
1k1j	-33.07	-1617.02	-1926.14	-102.66	-7.65	1.81
1jyr	-1038.98	-1744.62	-3036.58	-195.19	-8.22	0.28
1rlq	-222.49	-1576.62	-2330.39	-415.69	-9.56	0.64
2ke1	-71.83	-2361.52	-4251.26	-1124.31	-13.87	4.67
2bba	-740.01	-2916.44	-4692.84	-342.79	-9.12	6.38
1jgn	-1455.38	-2702.38	-5383.05	-416.08	-9.56	5.24
2roc	-2618.04	-2389.65	-6337.01	-520.12	-10.19	4.11

*(kcal/mol)

Vacuum/Shell 2*

System	$\Delta_f H$(Ligand)	$\Delta_f H$(Target)	$\Delta_f H$(Target:Ligand$[H_2O]_s$)	$\Delta_r H$	ΔH_b(calc)	|ε|
3ptb_ben	178.10	-1490.53	-1814.24	-155.01	-7.41	2.90
3ptb_pme	162.59	-1632.88	-1926.73	-167.43	-7.46	3.05
3ptb_pam	167.30	-1497.83	-1689.75	-128.02	-7.30	0.88
3ptb_pmo	130.65	-1718.48	-2100.65	-166.02	-7.46	3.71
3ptb_pad	136.51	-1672.10	-2290.93	-292.94	-7.98	5.04
1k1l	262.49	-1928.22	-2533.46	-289.74	-7.96	0.10
1k1m	19.38	-1688.03	-2627.09	-264.84	-7.86	0.36
1k1i	-117.59	-1957.77	-3113.13	-286.36	-7.95	2.95

1k1j	-33.07	-1617.02	-2666.43	-264.95	-7.86	1.60
1jyr	-1038.98	-1744.62	-4006.52	-413.72	-8.47	0.53
1rlq	-222.49	-1576.62	-4014.08	-769.98	-9.93	0.27
2ke1	-71.83	-2361.52	-5031.56	-1731.21	-13.86	4.66
2bba	-740.01	-2916.44	-4892.75	-369.30	-8.29	7.21
1jgn	-1455.38	-2702.38	-6921.86	-798.90	-10.05	4.75
2roc	-2618.04	-2389.65	-8129.03	-924.94	-10.56	3.74

*(kcal/mol)

Vacuum/Shell 3*

System	$\Delta_f H$(Ligand)	$\Delta_f H$(Target)	$\Delta_f H$(Target:Ligand[H_2O]$_s$)	$\Delta_r H$	ΔH_b(calc)	$\lvert\varepsilon\rvert$
3ptb_ben	178.10	-1490.53	-1894.35	-177.32	-6.44	1.93
3ptb_pme	162.59	-1632.88	-1994.60	-177.50	-6.44	2.03
3ptb_pam	167.30	-1497.83	-1904.57	-169.43	-6.39	0.03
3ptb_pmo	130.65	-1718.48	-2161.13	-168.70	-6.38	2.64
3ptb_pad	136.51	-1672.10	-2400.94	-287.35	-7.18	4.25
1k1l	262.49	-1928.22	-2906.69	-373.96	-7.77	0.10
1k1m	19.38	-1688.03	-2918.14	-324.68	-7.44	0.79
1k1i	-117.59	-1957.77	-3260.66	-318.30	-7.39	3.51
1k1j	-33.07	-1617.02	-2889.86	-314.97	-7.37	2.10
1jyr	-1038.98	-1744.62	-4063.15	-412.56	-8.03	0.09
1rlq	-222.49	-1576.62	-4143.39	-783.69	-10.53	0.33
2ke1	-71.83	-2361.52	-5363.26	-1369.31	-14.48	5.28
2bba	-740.01	-2916.44	-5814.11	-597.05	-9.27	6.23
1jgn	-1455.38	-2702.38	-7969.57	-1037.41	-12.24	2.56
2roc	-2618.04	-2389.65	-9169.78	-1156.49	-13.04	1.26

*(kcal/mol)

COSMO/Dry*

System	$\Delta_f H$(Ligand)	$\Delta_f H$(Target)	$\Delta_f H$(Target:Ligand[H_2O]$_s$)	$\Delta_r H$	ΔH_b(calc)	$\lvert\varepsilon\rvert$
3ptb_ben	121.51	-1586.93	-1524.91	-59.49	-8.23	3.73
3ptb_pme	110.35	-1747.18	-1655.03	-18.20	-4.95	0.53
3ptb_pam	111.93	-1588.82	-1513.72	-36.83	-6.43	0.01
3ptb_pmo	75.10	-1835.89	-1808.97	-48.18	-7.33	3.59
3ptb_pad	68.28	-1843.55	-1806.26	-31.00	-5.97	3.03
1k1l	105.03	-2055.90	-1973.22	-22.35	-5.28	2.59
1k1m	-45.84	-1848.02	-1919.22	-25.37	-5.52	2.71
1k1i	-187.90	-2072.55	-2318.30	-57.86	-8.10	2.80
1k1j	-92.20	-1757.78	-1917.79	-67.81	-8.89	0.57
1jyr	-1234.57	-1878.39	-3190.48	-77.52	-9.67	1.73
1rlq	-425.59	-1856.53	-2371.00	-88.88	-10.57	0.37
2ke1	-503.91	-2988.96	-3619.50	-126.63	-13.58	4.38
2bba	-919.44	-3156.63	-4172.38	-96.30	-11.16	4.34
1jgn	-1612.06	-2918.85	-4652.64	-121.72	-13.19	1.61
2roc	-2889.82	-2613.33	-5604.22	-101.07	-11.54	2.76

*(kcal/mol)

COSMO/Shell 1*

System	$\Delta_f H$(Ligand)	$\Delta_f H$(Target)	$\Delta_f H$(Target:Ligand[H_2O]$_s$)	$\Delta_r H$	ΔH_b(calc)	$\lvert\varepsilon\rvert$
3ptb_ben	121.51	-1586.93	-1597.54	-66.91	-6.84	2.33
3ptb_pme	110.35	-1747.18	-1733.93	-31.89	-5.53	1.12
3ptb_pam	111.93	-1588.82	-1728.52	-56.00	-6.43	0.02
3ptb_pmo	75.10	-1835.89	-1896.88	-70.87	-6.99	3.25
3ptb_pad	68.28	-1843.55	-1951.67	-45.99	-6.06	3.12
1k1l	105.03	-2055.90	-2343.04	-66.11	-6.81	1.05
1k1m	-45.84	-1848.02	-2227.18	-72.48	-7.05	1.17
1k1i	-187.90	-2072.55	-2469.51	-78.64	-7.28	3.62
1k1j	-92.20	-1757.78	-2113.17	-67.55	-6.86	2.60
1jyr	-1234.57	-1878.39	-3264.81	-86.64	-7.58	0.36
1rlq	-425.59	-1856.53	-2512.44	-99.90	-8.07	2.13
2ke1	-503.91	-2988.96	-4517.93	-242.54	-13.41	4.21
2bba	-919.44	-3156.63	-5100.12	-241.52	-13.37	2.13
1jgn	-1612.06	-2918.85	-5730.98	-287.11	-15.07	0.27
2roc	-2889.82	-2613.33	-6649.32	-233.22	-13.06	1.24

*(kcal/mol)

COSMO/Shell 2*

System	$\Delta_f H$(Ligand)	$\Delta_f H$(Target)	$\Delta_f H$(Target:Ligand[H_2O]$_s$)	$\Delta_r H$	ΔH_b(calc)	|ε|
3ptb_ben	121.51	-1586.93	-1947.84	-91.16	-6.96	2.45
3ptb_pme	110.35	-1747.18	-2047.97	-85.09	-6.89	2.48
3ptb_pam	111.93	-1588.82	-1819.98	-82.25	-6.86	0.44
3ptb_pmo	75.10	-1835.89	-2252.35	-100.29	-7.06	3.32
3ptb_pad	68.28	-1843.55	-2414.92	-117.97	-7.26	4.32
1k1l	105.03	-2055.90	-2725.01	-122.03	-7.30	0.56
1k1m	-45.84	-1848.02	-2813.28	-136.90	-7.47	0.75
1k1i	-187.90	-2072.55	-3293.99	-185.81	-8.02	2.88
1k1j	-92.20	-1757.78	-2881.42	-183.71	-7.99	1.47
1jyr	-1234.57	-1878.39	-4240.22	-214.31	-8.34	0.40
1rlq	-425.59	-1856.53	-4238.84	-326.46	-9.59	0.61
2ke1	-503.91	-2988.96	-5274.97	-803.95	-14.93	5.73
2bba	-919.44	-3156.63	-5321.09	-266.86	-8.92	6.58
1jgn	-1612.06	-2918.85	-7249.62	-501.55	-11.55	3.25
2roc	-2889.82	-2613.33	-8455.49	-474.33	-11.25	3.05

*(kcal/mol)

COSMO/Shell 3*

System	$\Delta_f H$(Ligand)	$\Delta_f H$(Target)	$\Delta_f H$(Target:Ligand[H_2O]$_s$)	$\Delta_r H$	ΔH_b(calc)	|ε|
3ptb_ben	121.51	-1586.93	-2039.70	-117.81	-5.69	1.18
3ptb_pme	110.35	-1747.18	-2130.87	-102.78	-5.36	0.95
3ptb_pam	111.93	-1588.82	-2042.38	-109.02	-5.50	0.92
3ptb_pmo	75.10	-1835.89	-2348.58	-131.31	-5.99	2.24
3ptb_pad	68.28	-1843.55	-2566.22	-138.85	-6.15	3.22
1k1l	105.03	-2055.90	-3126.41	-197.37	-7.44	0.43
1k1m	-45.84	-1848.02	-3101.83	-164.61	-6.72	1.50
1k1i	-187.90	-2072.55	-3441.60	-203.00	-7.56	3.34
1k1j	-92.20	-1757.78	-3077.59	-184.24	-7.15	2.32
1jyr	-1234.57	-1878.39	-4335.35	-244.23	-8.47	0.53
1rlq	-425.59	-1856.53	-4376.79	-333.99	-10.44	0.24
2ke1	-503.91	-2988.96	-5643.13	-389.58	-11.66	2.46
2bba	-919.44	-3156.63	-6229.64	-392.88	-11.73	3.77
1jgn	-1612.06	-2918.85	-8181.94	-520.91	-14.55	0.25
2roc	-2889.82	-2613.33	-9481.52	-587.42	-16.01	1.71

*(kcal/mol)

Vaccuum*

System	$\Delta_f H$
Water	-57.80

*(kcal/mol)

COSMO*

System	$\Delta_f H$
Water	-65.21

*(kcal/mol)

Supplementary Table 5 Linear regressions re-calculated without 2ke1

Per-system residuals (ε) and statistical parameters of linear regressions obtained with different water models

System	Vacuum				COSMO				
	Dry	Shell 1	Shell 2	Shell 3	Dry	Shell 1	Shell 2	Shell 3	Shell 3[b]
	|ε|[a]								
3ptb_ben	3.15	1.94	1.64	1.34	4.01	2.37	1.30	1.22	0.74
3ptb_pme	2.54	1.38	1.88	1.44	0.04	0.93	1.24	0.96	1.12
3ptb_pam	1.06	0.11	0.57	0.65	0.17	0.02	0.83	0.90	2.93
3ptb_pmo	3.88	2.58	2.53	2.02	3.64	3.31	2.29	2.29	0.46
3ptb_pad	6.12	4.77	4.77	4.05	2.72	3.02	3.53	3.27	1.51
1k1l	0.59	0.55	0.20	0.01	3.07	1.02	1.29	0.30	1.55
1k1m	1.08	1.85	0.84	0.85	3.13	1.10	1.28	1.42	2.95
1k1i	3.16	4.38	3.27	3.60	2.55	3.51	2.75	3.21	4.40
1k1j	2.44	3.14	2.08	2.20	0.11	2.56	1.37	2.21	3.57
1jyr	1.62	0.35	1.12	0.33	2.38	0.20	0.91	0.70	0.13
1rlq	2.36	2.76	2.87	1.89	1.26	1.88	1.43	0.52	0.49
2bba	7.69	4.09	6.94	5.33	3.29	0.96	5.34	3.42	2.93
1jgn	4.49	1.83	1.41	0.10	0.04	1.74	1.17	0.23	1.87
2roc	2.45	0.87	0.51	1.63	1.61	0.12	0.99	2.27	4.50
R^2	0.19	0.57	0.50	0.65	0.63	0.75	0.69	0.76	0.93
R^2(cv)[c]	0.03	0.45	0.39	0.56	0.52	0.68	0.62	0.67	0.91
F	2.79	15.76	12.03	22.29	20.15	36.47	27.23	37.41	173.54
RMSE[a]	3.88	2.83	3.05	2.55	2.63	2.14	2.38	2.12	2.61
t_α	1.67	3.97	3.47	4.72	4.49	6.04	5.22	6.12	13.17
t_β	-3.59	-3.04	-2.99	-3.37	-1.66	-4.06	-3.04	-2.77	-

[a]Unit: kcalmol^{-1}.

[b]Linear regression with β=0 (last column). and β≠0 (other columns).

[c]Leave-one-out cross-validated coefficient of determination.

Detailed results for three models

β≠0

Parameters of Eq. 3

Model	α	Δα*	β	Δβ*
Vacuum/Dry	1.6204×10^{-2}	9.6984×10^{-3}	-6.2967	1.7530
COSMO/Dry	1.0029×10^{-1}	2.2342×10^{-2}	-2.5495	1.5319
COSMO/Shell 3	2.3097×10^{-2}	3.7761×10^{-3}	-3.0011	1.0851

*Standard error of regression coefficient.

Vacuum/Dry*

System	ΔH_b(calc)	|ε|
3ptb_ben	-7.65	3.15
3ptb_pme	-6.95	2.54
3ptb_pam	-7.48	1.06
3ptb_pmo	-7.62	3.88

3ptb_pad	-9.05	6.12
1k1l	-8.45	0.59
1k1m	-7.14	1.08
1k1i	-7.74	3.16
1k1j	-7.02	2.44
1jyr	-9.56	1.62
1rlq	-12.56	2.36
2bba	-7.81	7.69
1jgn	-10.31	4.49
2roc	-11.85	2.45
**R^2**		**0.19**
**F**		**2.79**
**RMSE**		**3.88**

*(kcal/mol)

COSMO/Dry*

System	ΔH_b(calc)	|ε|
3ptb_ben	-8.52	4.01
3ptb_pme	-4.37	0.04
3ptb_pam	-6.24	0.17
3ptb_pmo	-7.38	3.64
3ptb_pad	-5.66	2.72
1k1l	-4.79	3.07
1k1m	-5.09	3.13
1k1i	-8.35	2.55
1k1j	-9.35	0.11
1jyr	-10.32	2.38
1rlq	-11.46	1.26
2bba	-12.21	3.29
1jgn	-14.76	0.04
2roc	-12.69	1.61
**R^2**		**0.63**
**F**		**20.15**
**RMSE**		**2.63**

*(kcal/mol)

COSMO/ Shell 3*

System	ΔH_b(calc)	|ε|
3ptb_ben	-5.72	1.22
3ptb_pme	-5.38	0.96
3ptb_pam	-5.52	0.90
3ptb_pmo	-6.03	2.29
3ptb_pad	-6.21	3.27
1k1l	-7.56	0.30
1k1m	-6.80	1.42
1k1i	-7.69	3.21
1k1j	-7.26	2.21
1jyr	-8.64	0.70
1rlq	-10.72	0.52
2bba	-12.08	3.42
1jgn	-15.03	0.23
2roc	-16.57	2.27
**R^2**		**0.76**
**F**		**37.41**
**RMSE**		**2.12**

*(kcal/mol)

β=0

Parameters of Eq. 3

Model	α	Δα*	β	Δβ*
Vacuum/Dry	4.4281×10^{-2}	7.9453×10^{-3}	0	-
COSMO/Dry	1.3332×10^{-1}	1.0941×10^{-2}	0	-
COSMO/Shell 3	3.1997×10^{-2}	2.4289×10^{-3}	0	-

*Standard error of regression coefficient.

Vacuum/Dry*

System	ΔH$_b$(calc)	|ε|
3ptb_ben	-3.70	0.80
3ptb_pme	-1.79	2.62
3ptb_pam	-3.24	3.18
3ptb_pmo	-3.61	0.13
3ptb_pad	-7.54	4.60
1k1l	-5.88	1.98
1k1m	-2.31	5.92
1k1i	-3.95	6.94
1k1j	-1.99	7.47
1jyr	-8.91	0.97
1rlq	-17.11	6.91
2bba	-4.13	11.37
1jgn	-10.96	3.84
2roc	-15.18	0.88
**R^2**		**0.70**
**R^2(cv)**		**0.71**
**F**		**31.06**
**RMSE**		**5.37**

*(kcal/mol)

COSMO/Dry*

System	ΔH$_b$(calc)	|ε|
3ptb_ben	-7.93	3.42
3ptb_pme	-2.43	1.99
3ptb_pam	-4.91	1.51
3ptb_pmo	-6.42	2.68
3ptb_pad	-4.13	1.20
1k1l	-2.98	4.88
1k1m	-3.38	4.84
1k1i	-7.71	3.18
1k1j	-9.04	0.42
1jyr	-10.34	2.40
1rlq	-11.85	1.65
2bba	-12.84	2.66
1jgn	-16.23	1.43
2roc	-13.48	0.82
**R^2**		**0.92**
**R^2(cv)**		**0.91**
**F**		**148.48**
**RMSE**		**2.81**

*(kcal/mol)

COSMO/Shell3*

System	ΔH$_b$(calc)	|ε|
3ptb_ben	-3.77	0.74
3ptb_pme	-3.29	1.12
3ptb_pam	-3.49	2.93
3ptb_pmo	-4.20	0.46
3ptb_pad	-4.44	1.51
1k1l	-6.32	1.55
1k1m	-5.27	2.95
1k1i	-6.50	4.40
1k1j	-5.90	3.57
1jyr	-7.81	0.13
1rlq	-10.69	0.49
2bba	-12.57	2.93
1jgn	-16.67	1.87
2roc	-18.80	4.50
**R^2**		**0.93**
**R^2(cv)**		**0.91**
**F**		**173.54**
**RMSE**		**2.61**

*(kcal/mol)

Supplementary Table 6 Linear regression (COSMO/Shell 3) re-calculated using two sub-sets

β ≠ 0

Parameters of Eq. 3

Sub-set	α	β
1	5.7500×10^{-2}	2.1228
2	1.9629×10^{-2}	-3.9127

COSMO/Shell 3*

System	ΔH_b(calc)	|ε|
3ptb_ben	-4.65	0.14
3ptb_pme	-3.79	0.62
3ptb_pam	-4.15	2.27
3ptb_pmo	-5.43	1.69
3ptb_pad	-5.86	2.93
1k1l	-9.23	1.36
1k1m	-7.34	0.88
1k1i	-9.55	1.35
1k1j	-8.47	0.99
1jyr	-8.71	0.77
1rlq	-10.47	0.27
2ke1	-11.56	2.36
2bba	-11.62	3.88
1jgn	-14.14	0.66
2roc	-15.44	1.14
**R^2**		**0.80**
**F**		**50.91**
**RMSE**		**1.67**

*(kcal/mol)

β = 0

Parameters of Eq. 3

Sub-set	α	β
1	4.4122×10^{-2}	-
2	2.8461×10^{-2}	-

COSMO/Shell 3*

System	ΔH_b(calc)	|ε|
3ptb_ben	-5.20	0.69
3ptb_pme	-4.54	0.12
3ptb_pam	-4.81	1.61
3ptb_pmo	-5.79	2.05
3ptb_pad	-6.13	3.19
1k1l	-8.71	0.85
1k1m	-7.26	0.96
1k1i	-8.96	1.94
1k1j	-8.13	1.34
1jyr	-6.95	0.99
1rlq	-9.51	0.69
2ke1	-11.09	1.89
2bba	-11.18	4.32
1jgn	-14.83	0.03
2roc	-16.72	2.42
**R^2**		**0.96**
**F**		**336.60**
**RMSE**		**1.93**

*(kcal/mol)

Supplementary Table 7 Linear regressions of three models with β=0

Parameters of Eq. 3

Model	α	Δα*	β	Δβ*
Vacuum/Dry	2.2784×10^{-2}	6.3704×10^{-3}	0	-
COSMO/Dry	1.2143×10^{-1}	1.1437×10^{-2}	0	-
COSMO/Shell 3	3.1025×10^{-2}	2.3146×10^{-3}	0	-

*Standard error of regression coefficient.

Vacuum/Dry*

System	ΔH_b(calc)	|ε|
3ptb_ben	-1.91	2.60
3ptb_pme	-0.92	3.49
3ptb_pam	-1.66	4.75
3ptb_pmo	-1.86	1.88
3ptb_pad	-3.88	0.94
1k1l	-3.03	4.84
1k1m	-1.19	7.04
1k1i	-2.03	8.86
1k1j	-1.02	8.44
1jyr	-4.59	3.35
1rlq	-8.81	1.39
2ke1	-20.26	11.06
2bba	-2.13	13.37
1jgn	-5.64	9.16
2roc	-7.81	6.49
**R^2**		**0.48**
**R^2(cv)**		**0.27**
**F**		**12.79**
**RMSE**		**7.12**

*(kcal/mol)

COSMO/Dry*

System	ΔH_b(calc)	|ε|
3ptb_ben	-7.22	2.72
3ptb_pme	-2.21	2.20
3ptb_pam	-4.47	1.94
3ptb_pmo	-5.85	2.11
3ptb_pad	-3.76	0.83
1k1l	-2.71	5.15
1k1m	-3.08	5.14
1k1i	-7.03	3.87
1k1j	-8.23	1.23
1jyr	-9.41	1.47
1rlq	-10.79	0.59
2ke1	-15.38	6.18
2bba	-11.69	3.81
1jgn	-14.78	0.02
2roc	-12.27	2.03
**R^2**		**0.89**
**R^2(cv)**		**0.87**
**F**		**112.73**
**RMSE**		**3.27**

*(kcal/mol)

COSMO/Shell 3*

System	ΔH_b(calc)	|ε|
3ptb_ben	-3.66	0.85
3ptb_pme	-3.19	1.22
3ptb_pam	-3.38	3.03
3ptb_pmo	-4.07	0.33
3ptb_pad	-4.31	1.37
1k1l	-6.12	1.74
1k1m	-5.11	3.12

1k1i	-6.30	4.60
1k1j	-5.72	3.75
1jyr	-7.58	0.36
1rlq	-10.36	0.16
2ke1	-12.09	2.89
2bba	-12.19	3.31
1jgn	-16.16	1.36
2roc	-18.22	3.92
**R^2**		**0.93**
**R^2(cv)**		**0.91**
**F**		**179.66**
**RMSE**		**2.65**

*(kcal/mol)
